# EEG-Based Seizure Detection Using Dual-Branch CNN-ViT Network Integrating Phase and Power Spectrograms

**DOI:** 10.3390/brainsci15050509

**Published:** 2025-05-16

**Authors:** Zhuohan Wang, Yaoqi Hu, Qingyue Xin, Guanghao Jin, Yazhou Zhao, Weidong Zhou, Guoyang Liu

**Affiliations:** 1 School of Integrated Circuits, Shandong University, Jinan 250199, China; 202200201047@mail.sdu.edu.cn (Z.W.); 202432376@mail.sdu.edu.cn (Y.H.); 202432383@mail.sdu.edu.cn (Q.X.); 2 Institute of Computer Science, Ludwig Maximilian University of Munich, 80539 Munich, Germany; jin@cip.ifi.lmu.de; 3 Department of Biomedical Engineering, New York University, New York, NY 10012, USA; yz11003@nyu.edu; 4 Shenzhen Research Institute, Shandong University, Shenzhen 518000, China; 5 Yunnan Research Institute, Shandong University, Yunnan 650000, China

**Keywords:** seizure detection, continuous wavelet transform, convolutional neural network, vision transformer

## Abstract

**Background/Objectives:** Epilepsy is a common neurological disorder with pathological mechanisms closely associated with the spatiotemporal dynamic characteristics of electroencephalogram (EEG) signals. Although significant progress has been made in epileptic seizure detection methods using time–frequency analysis, current research still faces challenges in terms of an insufficient utilization of phase information. **Methods:** In this study, we propose an effective epileptic seizure detection framework based on continuous wavelet transform (CWT) and a hybrid network consisting of convolutional neural network (CNN) and vision transformer (ViT). First, the raw EEG signals are processed by the CWT. Then, the phase spectrogram and power spectrogram of the EEG are generated, and they are sent into the designed CNN and ViT branches of the network to extract more discriminative EEG features. Finally, the features output from the two branches are fused and fed into the classification network to obtain the detection results. **Results:** Experimental results on the CHB-MIT public dataset and our SH-SDU clinical dataset show that the proposed framework achieves sensitivities of 98.09% and 89.02%, specificities of 98.21% and 95.46%, and average accuracies of 98.45% and 94.66%, respectively. Furthermore, we compared the spectral characteristics of CWT with other time–frequency transforms within the hybrid architecture, demonstrating the advantages of the CWT-based CNN-ViT architecture. **Conclusions:** These results highlight the outstanding epileptic seizure detection performance of the proposed framework and its significant clinical feasibility.

## 1. Introduction

Epilepsy is a chronic neurological disorder characterized by abnormal synchronous discharge of neurons in the brain. It affects approximately 50 million people worldwide, with about 5 million new cases each year and a lifetime prevalence of 7.6 per 1000 individuals [1,2]. Clinically, epilepsy manifests as recurrent tonic–clonic seizures or absence seizures, often accompanied by cognitive dysfunction [3]. In addition, psychiatric comorbidities such as depression and anxiety are common among individuals with epilepsy, with a bidirectional relationship between psychiatric symptoms and seizure frequency [4,5]. For patients with drug-resistant epilepsy, the risk of sudden unexpected death in epilepsy is significantly elevated, likely due to autonomic dysregulation and brainstem dysfunction [6,7].

Electroencephalography (EEG) is a cornerstone tool for the diagnosis and treatment of epilepsy. It records the synchronized discharge activity of neuronal populations through scalp or intracranial electrodes, enabling precise detection of brain function changes across both temporal and spatial dimensions [8]. This spatiotemporal characterization of neural activity forms the physiological basis for clinicians to detect epileptiform patterns through EEG signal analysis [9]. EEG plays a critical role in distinguishing the nature of seizures and guiding the selection of appropriate antiepileptic drugs [10]. High-frequency oscillations and interictal epileptiform discharges have become key biomarkers for the localization of epileptogenic foci in presurgical assessments, as they exhibit distinct spatiotemporal characteristics that are crucial for surgical planning [11]. Additionally, long-term EEG monitoring offers a reliable means for the clinical early warning of critical situations. However, traditional EEG analysis is heavily reliant on the expertise of neurologists, and inter-rater agreement among different clinicians is often low [12]. Therefore, the development of automated seizure detection systems based on EEG is imperative to enhance diagnostic accuracy and efficiency.

In early studies, epileptic EEG pattern classification heavily relied on manual feature extraction alongside classical machine learning methods. The spike-wave detection algorithm [13], which involves the manual extraction of morphological features such as waveform amplitude and spike duration, laid the foundation for traditional analysis techniques. Subsequently, a series of machine learning-based algorithms gradually matured. Researchers employed Bayesian linear discriminant analysis (BLDA) to construct classification models, achieving a sensitivity of up to 96.25% [14]. By incorporating nonlinear dynamic features such as correlation dimension and multiscale entropy, and using support vector machines (SVMs) for classification, the sensitivity for epileptic focus localization was significantly improved [15]. Furthermore, the fusion of circadian rhythm features with logistic regression models effectively reduced artifact interference while achieving high accuracy [16]. Although traditional clinical epilepsy classification methods rely on subjective expert interpretation of EEG waveform characteristics such as spike frequency and morphological features, combined with empirical pathological correlations, the long-term clinical accumulation of spectral characteristics and dynamic feature systems in waveforms has established an interpretable mathematical foundation for machine learning models [17].

Time–frequency analysis methods provide a multiscale feature representation framework by jointly characterizing the temporal evolution and frequency distribution of signals, offering a comprehensive approach for complex pattern analysis. In traditional methods, short-time Fourier transform (STFT) is constrained by the time–frequency resolution trade-off imposed by the fixed window function [18,19], making it difficult to capture the transient characteristics of non-stationary signals. The Stockwell transform (S-transform) introduces frequency adaptability by employing a time-varying Gaussian window, improving resolution in the low-frequency range. However, its time–frequency focus remains insufficient in the high-frequency range, and its computational complexity significantly increases [20,21]. The Synchrosqueezing transform (SST) enhances noise robustness by reorganizing the time–frequency energy spectrum, but due to its dependence on the initial STFT spectrum, it is unable to fully overcome the constraints imposed by the window function [22,23]. These limitations of traditional time–frequency methods are particularly evident when dealing with non-stationary signals, where the frequency components change dynamically over time [24]. In contrast, continuous wavelet transform (CWT) utilizes basis functions with both time and frequency localization properties, allowing for narrow time windows in the high-frequency range to capture instantaneous details, while using wide time windows in the low-frequency range to preserve the overall trend of the signal. This makes CWT particularly suitable for processing non-stationary signals with prominent dynamic variations [25,26]. Existing studies have demonstrated that CWT-based time–frequency representations are highly compatible with deep learning networks, exhibiting excellent performance in non-stationary signal analysis tasks across various domains [27,28,29].

In recent years, the Transformer model has garnered significant attention in the field of Natural Language Processing (NLP), with its core mechanism, self-attention, effectively capturing long-range dependencies and global features [30]. In the domain of image analysis, by serializing the segmented image patches and inputting them into the Transformer, the vision transformer (ViT) was introduced, achieving remarkable breakthroughs in image classification tasks [31]. Meanwhile, the convolutional neural network (CNN), with its ability to extract local features and inherent translational invariance, remains the dominant approach in image recognition [32]. Moreover, multi-branch fusion strategies have been extensively validated in medical image analysis. For instance, the U-Net architecture has significantly improved segmentation accuracy in biomedical image segmentation [33], and the combination of spatial information enhancement with boundary shape correction has notably enhanced multimodal MRI segmentation performance [34].

With the advancement of research on epileptic seizure detection based on time–frequency representations of EEG signals, the potential value of phase information has gradually been revealed [21]. However, the choice of time–frequency transform methods may impact the complementary effects of EEG phase and amplitude information, and the differences in the representational capabilities of various deep network architectures for time–frequency features have not been thoroughly examined. To this end, we compared the results of phase-power spectrograms generated by three time–frequency analysis methods, CWT, STFT, and S transform, after the hybrid network. Furthermore, we analyzed the advantages of CNN, ViT, and their hybrid architectures in extracting these two types of features. We then propose in this paper a CNN-ViT hybrid network that is capable of integrating heterogeneous features, demonstrating its synergistic effect in combining local texture sensitivity with global dependency modeling. Finally, we present an epileptic seizure detection system based on CWT spectrograms and a CNN-ViT hybrid deep network. The main contributions of this study are as follows:

- We propose a dual-branch CNN-ViT hybrid network based on the phase and power spectrogram derived from CWT, enabling the complementary representation of time–frequency features and resulting in a significant improvement in seizure detection performance.
- We systematically reveal the sensitivity of CNN to the phase spectrogram and the modeling advantages of ViT for the power spectrogram, demonstrating the rationality of the network design.
- We evaluate the proposed network on the public CHB-MIT database and our clinically collected SH-SDU database. The proposed seizure detection framework demonstrates excellent performance in terms of sensitivity, specificity, and accuracy, showing its clinical generalization potential.

## 2. EEG Database

### 2.1. CHB-MIT Database

The first dataset used in this study is the CHB-MIT dataset [35], which is widely used in epilepsy seizure detection and EEG analysis research. This dataset comprises multi-channel EEG recordings from 24 epilepsy patients, collected at Boston Children’s Hospital, totaling approximately 980 h. EEG data were captured at a sampling rate of 256 Hz with 16-bit resolution using 18 to 23 bipolar electrodes arranged according to the international 10–20 electrode placement standard. Each EEG segment includes distinct seizure and non-seizure intervals, meticulously annotated by clinical experts with precise onset and offset timestamps. Overall, the dataset documents 184 annotated epileptic seizure events, with 40 seizures specifically designated for training purposes. To ensure both consistency and comparability across EEG recordings, this study employs 18 common bipolar electrode channels in the CHB-MIT database, including Fp1-F7, F7-T7, T7-P7, P7-O1, Fp1-F3, F3-C3, C3-P3, P3-O1, Fp2-F4, F4-C4, C4-P4, P4-O2, Fp2-F8, F8-T8, T8-P8, P8-O2, Fz-Cz, and Cz-Pz. Further details on this dataset are summarized in Table 1.

### 2.2. SH-SDU Database

This study also includes a clinical scalp EEG dataset from the Second Hospital of Shandong University (SH-SDU). The SH-SDU dataset contains continuous, long-duration EEG monitoring data from six adult epilepsy patients, recorded at a sampling frequency of 500 Hz. All recordings were acquired using a Natus NicoletOne EEG system (Nicolet v32 amplifier, Natus Medical Inc., San Carlos, CA, USA) with data stored in the EDF format. The scalp electrodes were placed according to the standard 10–20 system protocol. EEG signals were captured using an 18-channel monopolar configuration, with the reference electrode positioned centrally between Fz and Cz. Electrode sites included standard scalp locations: Fp1, Fp2, F3, F4, C3, P3, P4, O1, O2, F7, F8, T3, T4, T5, T6, A1, and A2. The data were not manually pre-selected, and clinical experts conducted a retrospective analysis of a total of 87.55 h of continuous recordings, annotating 97 seizure events. Compared to the CHB-MIT dataset, the SH-SDU dataset has the following characteristics: (1) the patient group consists of adults aged 28–79 years, with a wide age range; and (2) the seizure frequency is significantly higher, providing denser pathological samples for studying seizure-related features. A more detailed description of this dataset can be found in Table 2.

## 3. Method

**Figure 1:**
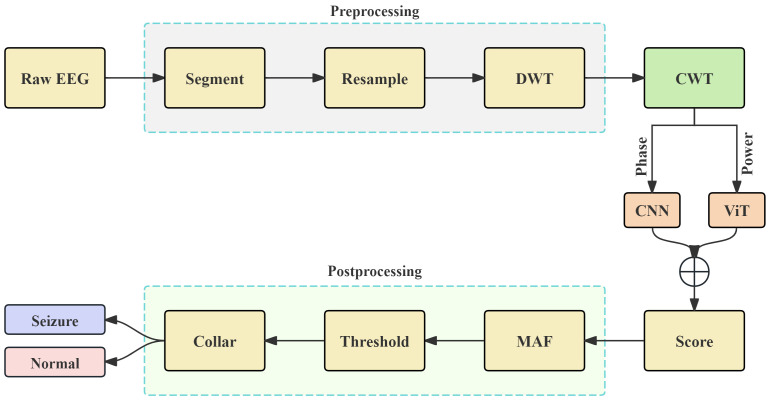
The workflow of the proposed epilepsy detection architecture is illustrated. The preprocessing module includes signal segmentation, EEG resampling, and discrete wavelet transform (DWT). After CWT, the deep learning network consists of the Phase-CNN and Power-ViT modules. The postprocessing module includes moving average filter (MAF), score thresholding, and the Collar Technique.

As shown in Figure 1, the proposed seizure detection system can be roughly divided into four main modules: preprocessing, time–frequency transform, deep learning models, and postprocessing. Each module will be discussed in detail in the following sections.

### 3.1. Preprocessing

For epileptic seizure detection and model training, the data from the CHB-MIT dataset were preprocessed to remove artifacts caused by factors such as electrooculography (EOG) and electromyography (EMG) [35]. Researchers commonly apply DWT to remove high-frequency noise and focus on signals in the seizure frequency band. Previous studies have shown that DWT with the Db4 wavelet has been successfully applied to EEG classification for seizure detection [36,37]. The raw EEG signals were first decomposed into 4-s EEG segments, resulting in non-overlapping 4-s multi-channel EEG segments with 1024 points. DWT decomposes each EEG segment into five scales corresponding to 64–128 Hz (D1), 32–64 Hz (D2), 16–32 Hz (D3), 8–16 Hz (D4), and 4–8 Hz (D5). Additionally, an approximation term corresponding to 0–4 Hz (A5) is generated. Given that the epileptiform mainly exists within the 3–29 Hz range [38], we selected the D3, D4, and D5 scales to reconstruct the 4–32 Hz frequency band for further analysis. Afterward, each 4-s segment was processed using CWT based on the complex Morlet wavelet to obtain the power and phase spectrogram [39]. For each 4-s segment, a total of 36 spectra can be obtained from the 18 EEG channels. These CWT spectrograms are then used to train deep learning models to provide the final prediction results.

### 3.2. CWT with Complex Morlet Wavelet

For a time-domain signal, CWT is defined as

$$\mathrm{CWT}\left(a,b\right)=\frac{1}{\sqrt{a}}\int_{-\infty }^{+\infty }x\left(t\right)\;\psi^{*}\left(\frac{t-b}{a}\right)dt$$

where *a* is the scale parameter that governs the dilation of the wavelet; a larger *a* results in a broader wavelet, capturing lower-frequency components, while a smaller *a* yields a narrower wavelet, capturing higher-frequency details. The parameter *b* represents the translation factor, determining the wavelet’s position along the time axis. The complex Morlet wavelet is a widely used complex-valued wavelet, commonly applied in time–frequency analysis. Its mother wavelet function is mathematically defined as

$$\psi \left(t\right)=\frac{1}{\sqrt{\pi f_{b}}}\;e^{2\pi if_{c}t}\;e^{-t^{2}/f_{b}}$$

where $f_{b}$ is the bandwidth parameter that controls the wavelet’s temporal width and frequency resolution, and $f_{c}$ is the center frequency, which determines the primary oscillation frequency of the wavelet. Substituting the complex Morlet wavelet into the CWT yields

$$W_{x}\left(a,b\right)=\frac{1}{\sqrt{a\pi f_{b}}}\int_{-\infty }^{+\infty }x\left(t\right)\;e^{-2\pi if_{c}(\frac{t-b}{a})}\;e^{-{\left(\frac{t-b}{a}\right)}^{2}/f_{b}}\;dt$$

In this study, we set $f_{b}=1$ and $f_{c}=1$ to balance time and frequency resolution. Given x(t), the CWT can be written as

$$W_{x}\left(a,b\right)=A\left(a,b\right)\;e^{i\phi (a,b)}$$

The power spectrogram of the CWT can be calculated using the following formula:

$${\left|W_{x}\left(a,b\right)\right|}^{2}=W_{x}\left(a,b\right)\cdot W_{x}^{*}\left(a,b\right)$$

The corresponding phase spectrogram can be derived through the equation presented below:

ϕ(a,b)=arctanIm(Wx(a,b))Re(Wx(a,b))

**Figure 2:**
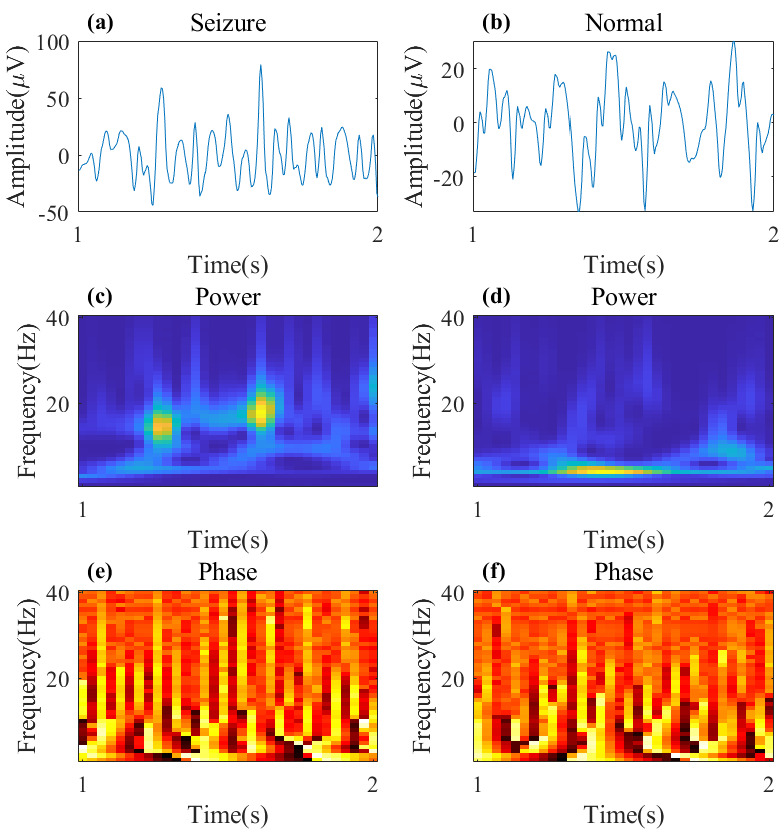
Time-domain and time–frequency-domain representations of both epileptic segment and normal segment. (**a**,**b**) The time-domain representation over the time range of 1–2 s. (**c**,**d**) The power spectrogram obtained after applying the CWT, with the y-axis representing the frequency range (0–40 Hz). (**e**,**f**) The phase spectrogram derived from the CWT, with the y-axis representing the frequency range (0–40 Hz).

Figure 2 illustrates sample segments of both epileptic and normal EEG signals, showing the time-domain EEG signals and their CWT representations.

### 3.3. Hybrid CNN-ViT Architecture for Seizure Detection

**Figure 3:**
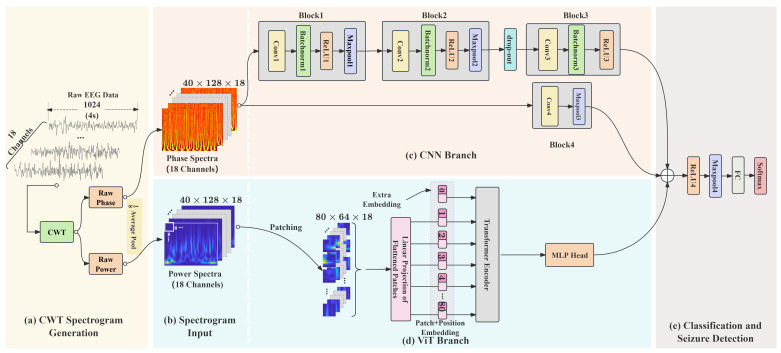
A schematic diagram of the hybrid architecture. (**a**) The CWT spectrogram generation with an average pooling layer. (**b**) The input of spectrograms. (**c**) The CNN branch with a deeper refinement path consists of three basic blocks and a dropout layer. The shortcut path is composed of a simplified block. (**d**) The ViT branch. The power spectrum feature map is divided and flattened before being passed into the linear projection layer. And the sequence is input into the Transformer encoder after adding a learnable positional encoding. (**e**) The classification is accomplished through the integration of a ReLU layer, a max pooling layer, an FC layer, and a softmax layer.

This study proposes a dual-branch hybrid network architecture, which integrates the CWT representation of EEG signals with deep learning networks. The first branch of the network is designed as a CNN with shortcut connections, where the input is the phase spectrogram generated by the CWT. This branch extracts time–frequency phase features of the EEG signals through multiple convolutional layers. Previous works demonstrate that the residual structure effectively mitigates the vanishing gradient problem. The second branch utilizes a single-layer ViT structure, with the input being the power spectrogram. This branch effectively models the global energy distribution characteristics of the power spectrogram, capturing power relationships across frequency bands. To mitigate the computational redundancy caused by high temporal resolution in the raw CWT spectrum, we first apply an average pooling operation with a kernel size of 8 and a stride of 8 along the temporal axis (shown in Figure 3a). This operation downsamples the temporal dimension from the original 1024 points to 128 points, effectively reducing the temporal dimensionality while preserving critical power and phase information.

#### 3.3.1. CNN with Shortcut Based on Phase Spectrogram

The architecture of the proposed CNN branch is shown in Figure 3c. The CNN branch takes the phase spectrogram matrix $I_{\mathrm{CWT}}\in R^{40\times 128\times 18}$ as input and employs a dual-path parallel architecture for multiscale feature extraction. The deeper structure consists of three convolutional blocks. The first two blocks are composed of 3×3 convolutions with a single stride, batch normalization (BatchNorm), ReLU activation, and 2×2 max pooling with a stride length of 2. These blocks progressively reduce the dimensionality of the features. Additionally, a dropout layer (rate = 0.2) is introduced after the second block to prevent overfitting [40]. The third block removes the pooling layer, retaining only the convolution and ReLU layers to avoid the loss of high-frequency details. The shortcut path consists of a single 3×3 convolution (stride 1) and a 4×4 max-pooling layer (stride 4), which rapidly compresses the spatial dimensions and captures global trend features over a wide temporal window. The outputs of the two paths are concatenated along the channel dimension to integrate multiscale information, which is then passed directly to the subsequent classifier for learning.

#### 3.3.2. ViT Based on Power Spectrogram

The architecture of the ViT branch in this study is shown in Figure 3d. The pooled spectrogram matrix $I_{\mathrm{CWT}}\in R^{40\times 128\times 18}$ is then divided into non-overlapping patches $I_{p}\in R^{80\times (8^{2}\times 18)}$ of equal size, where 8 represents the size of each image patch and $80=40\times 128/8^{2}$ represents the number of image patches. The image patch embeddings are obtained by applying a linear transformation, which maps each flattened image patch vector to a *d*-dimensional representation. A learnable class embedding vector is added at the beginning of the image patch embedding sequence to introduce a global classification feature. To preserve the spatial relationships of the image patches in the original image, ViT employs a learnable *1-D* positional encoding that integrates positional information into the image patch embeddings. The resulting sequence of embedding vectors is then fed into the encoder as input.

**Figure 4:**
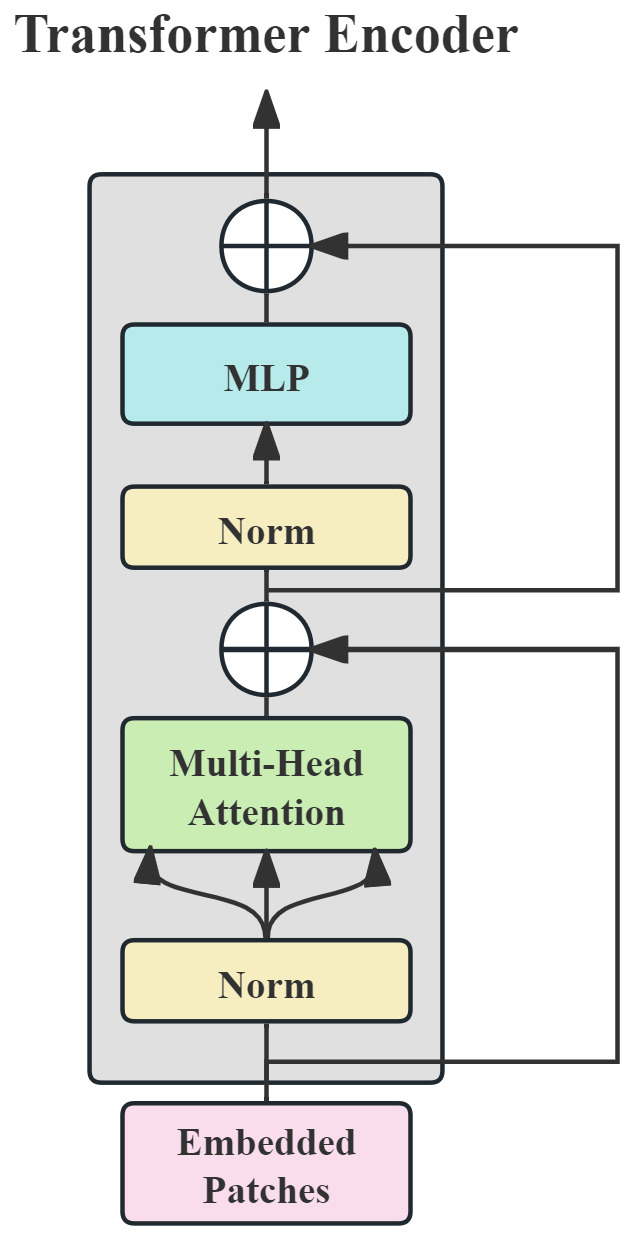
The architecture of the Transformer encoder. It consists of a normalization layer, multi-head attention layers, and a multi-layer perceptron.

The Transfomer encoder is shown in Figure 4. The encoder typically consists of multi-head attention (MHA) and multi-layer perceptron (MLP) mechanisms, which are used to further process the embedding vectors and extract features to generate the final feature representation. Given the input sequence embedding vectors (where *N* is the sequence length and *d* is the feature dimension), MHA projects the input into *h* independent sets of Query, Key, and Value matrices:

$${\mathrm{head}}_{i}=\mathrm{softmax}\left(\frac{\left(XW_{i}^{Q}\right){\left(XW_{i}^{K}\right)}^{⊤}}{\sqrt{d/h}}\right)\left(XW_{i}^{V}\right)$$

where $W_{i}^{Q},W_{i}^{K},W_{i}^{V}\in R^{d\times d/h}$ represents the learnable parameters, and *h* is the number of attention heads. The outputs of all attention heads are concatenated and then linearly transformed to obtain the final attention features:

$$\mathrm{MHA}\left(X\right)=\mathrm{Concat}\left\{{\mathrm{head}}_{1},\ldots ,{\mathrm{head}}_{h}\right\}W^{O}$$

This mechanism learns the associated weights of different subspaces in parallel, effectively modeling the global energy correlation across frequency bands in the power spectrogram. The MLP performs nonlinear transformations and dimensional expansion on the attention features:

$$\mathrm{MLP}\left(Z\right)=\mathrm{GELU}\left(ZW_{1}+b_{1}\right)W_{2}+b_{2}$$

Here, $W_{1}\in R^{d\times 4d},W_{2}\in R^{4d\times d}$ represents the parameters of the fully connected layer, and the GELU activation function [41] enhances the model’s ability to fit nonlinear relationships. Additionally, the output of each submodule is passed through a residual connection followed by layer normalization (LayerNorm):

$$\begin{array}{cc} Z^{\prime } & =\mathrm{LayerNorm}\left(X+\mathrm{MHA}(X)\right)\\ X_{\mathrm{out}} & =\mathrm{LayerNorm}\left(Z^{\prime }+\mathrm{MLP}\left(Z^{\prime }\right)\right) \end{array}$$

which helps to effectively mitigate the vanishing gradient problem and stabilize the training process [42].

In this study, the single-layer ViT encoder directly processes the power spectrogram time–frequency image sequence generated by the CWT. Through the aforementioned mechanism, it extracts global cross-frequency features related to epilepsy, which complement the local phase features extracted by the CNN branch. The outputs of the CNN and ViT branches are concatenated along the channel dimension and passed through a ReLU activation function to introduce nonlinear transformations, enhancing feature separability. A 4 × 4 max pooling operation with a stride length of 4 is then applied to further compress the feature map size, preserving significant responses while reducing computational complexity. The pooled output is flattened and passed into a fully connected (FC) layer, mapping it to a two-dimensional vector, which is finally processed by a softmax function to generate the probability distribution for the Seizure and Normal classes as shown in Figure 3e.

### 3.4. Model Training

In the CHB-MIT dataset, 184 epileptic seizure events are labeled for training and testing, with 40 used for training and the remainder for testing. The first seizure event of most patients is used as training data, which aligns with real-world scenarios where the available ictal data for each patient is typically limited in the early stage, while for patients with a higher frequency or short length of seizures (e.g., Patients 6, 12, 13, and 16), the first four to eight seizure events are selected. To compensate for the limited training data, each seizure event is overlapped five times to balance the training and testing datasets. Ultimately, 3.04 h of recordings are used for training, and the remaining 976.89 h are used for testing. In the SH-SDU dataset, 109 epileptic seizure events are labeled for training and testing, with 12 used for training and the remainder for testing.

The training was performed using the Adam optimizer [43] for end-to-end training, with an initial learning rate set to $2\times 10^{-4}$, a maximum of 100 training epochs, and an exponential decay factor $\gamma ={\left(2\times 10^{-5}/2\times 10^{-4}\right)}^{0.2}$ applied every 20 epochs, ultimately reducing the learning rate to $2\times 10^{-5}$. This strategy balances rapid convergence during the early stages of training with fine-tuning in the later stages [44]. L2 regularization with weight decay (coefficient $1\times 10^{-3}$) was introduced to suppress overfitting. The batch size was set to 128 to accommodate GPU memory limitations while ensuring the stability of gradient estimates [45].

### 3.5. Postprocessing

**Figure 5:**
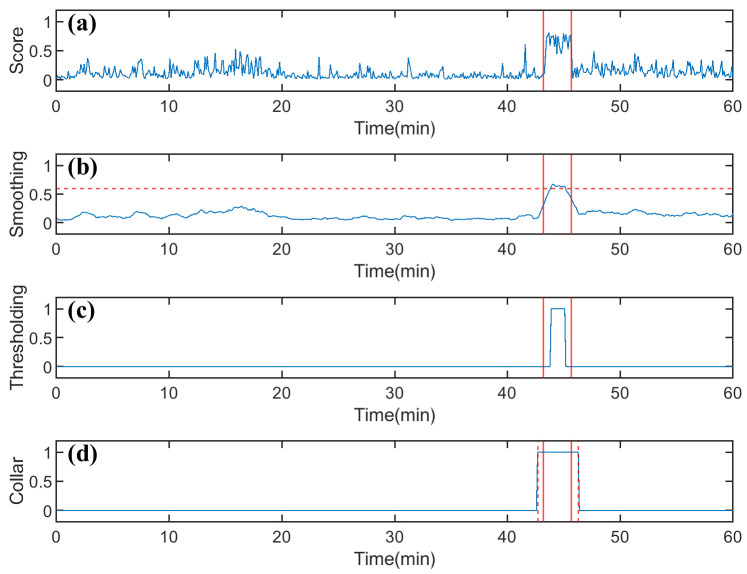
Postprocessing procedure for the output. (**a**) Score Summation. The output scores of four consecutive segments are summed together. The red solid line indicates the seizure interval annotated by experts. (**b**) Smoothing. A sliding average filter is applied to smooth the total score over time. (**c**) Thresholding. The smoothed scores are compared with a predefined threshold (red dashed line) to generate a series of binary decisions (Seizure = 1, Normal = 0). (**d**) Collar. The Collar Technique is used to compensate for the phase delay introduced by the smoothing filter, yielding the final labels. The seizure boundaries on both sides are extended by 12 time points.

To mitigate the transient variability in model output scores and improve seizure detection performance, we designed a postprocessing pipeline [46], as shown in Figure 5. First, the model output probabilities of four consecutive 4-s EEG segments are summed to expand the total score range to [−4,+4], enhancing the temporal continuity of seizure events. A moving average filter (MAF) with a window length of 2N+1(N=3) is then applied to smooth the total score. The mathematical expression for this process is given as

$$S\left(t\right)=\frac{1}{2N+1}\sum_{k=-N}^{N}x\left(t+k\right)$$

This operation effectively suppresses random fluctuations and reduces the false detection rate. Subsequently, the smoothed scores are compared with the patient-specific adaptive threshold *Thr*. Segments with scores above *Thr* are labeled as seizure events (1), while those below *Thr* are labeled as normal (0). The *Thr* is optimized based on individual data to balance sensitivity and specificity. To avoid the blurring of seizure onset or termination points due to smoothing, the Collar Technique is further applied, extending the detected seizure segments by 12 points on both sides, ensuring that the clinically relevant transition areas are fully covered.

### 3.6. Performance Metrics and Evaluation Setup

All experiments were conducted in MATLAB R2024b, operating on a PC equipped with an Intel Core i9-13900K 3.00 GHz CPU and an NVIDIA GeForce RTX 3090 GPU. Preprocessing and DWT were performed on the CPU, while CNN training and prediction were executed on the GPU. This study employs the following metrics to evaluate the model performance:

$$\begin{array}{cc} \mathrm{Sensitivity} & =\frac{\mathrm{TP}}{\mathrm{TP}+\mathrm{FN}}\times 100\%\\ \mathrm{Specificity} & =\frac{\mathrm{TN}}{\mathrm{TN}+\mathrm{FP}}\times 100\%\\ \mathrm{Accuracy} & =\frac{\mathrm{TP}+\mathrm{TN}}{\mathrm{TP}+\mathrm{TN}+\mathrm{FP}+\mathrm{FN}}\times 100\% \end{array}$$

In this study, TP, TN, FP, and FN refer to the number of true positive, true negative, false positive, and false negative segments, respectively. Sensitivity reflects the ratio of correctly identified seizure segments to the total number of seizure segments annotated by experts, while specificity measures the ratio of correctly identified normal segments. Accuracy indicates the ratio of correctly detected segments to the total number of EEG segments in the test dataset. Additionally, we adopted the false detection rate (FDR), which quantifies the proportion of false positive seizure events. This metric is defined as the average number of false positives per hour. To further assess the performance of the detection system, we computed the average Receiver Operating Characteristic (ROC) curve across all patients and calculated the Area Under the Curve (AUC) for each patient. The ROC curve, plotted by varying classification thresholds, uses the AUC value to quantify the model’s global ability to distinguish between seizure and non-seizure segments [47]. Typically, an AUC-ROC of 0.5 (50%) indicates a model with no discriminative power. In this study, we set the smoothing window length and collar length to 24 s when calculating the AUC-ROC. This metric, which is independent of the classification threshold and accounts for all possible error trade-offs [48], provides a more objective evaluation of the dataset.

## 4. Results

### 4.1. Results on CHB-MIT Database

The segment-based results for all patients on the CHB-MIT dataset are presented in Table 3. It can be observed that the model achieves excellent performance in the majority of patients, with an average sensitivity of 98.09% and an average specificity of 98.21%. Two-thirds of the patients reach 100% sensitivity, with specificity exceeding 98%, and the accuracy of 16 patients exceeds 99%.

Table 4 presents the event-based detection performance evaluation results. In the clinical EEG environment, which is characterized by significant individual variability and complex noise interference, the proposed method demonstrates exceptional performance. Among 24 epileptic patients, 22 patients (91.67%) achieved a 100% seizure event detection rate (all seizure events were accurately labeled), with the overall average event detection sensitivity reaching 98.95%. Additionally, in terms of false detection rate control, 19 patients (79.17%) maintained an FDR consistently below 0.5 events per hour. These satisfactory results also indicate that the proposed seizure detection method performs well on patients with various seizure types, further validating the generalization ability of the proposed method. Furthermore, the latency results, as listed in Table 4, shows the average time difference between the seizure onset labeled by experts and the system-detected onset for each patient. The reported latency for all patients is negative, which suggests that the proposed detection model successfully predicts seizures ahead of the expert annotations. On average, the latency is below −20 s, demonstrating the system’s ability to anticipate seizures effectively before they occur.

**Figure 6:**
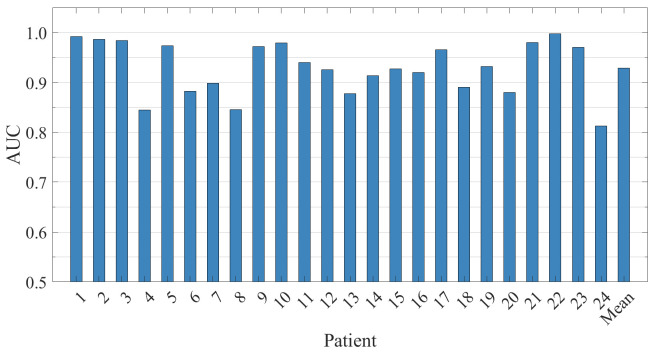
The ROC-AUC results for 24 patients in the CHB-MIT dataset are evaluated and presented along with the average values.

The proposed method demonstrates excellent and stable classification performance in an independent test across 24 patients, as shown in Figure 6. The AUC for all cases exceeds 80%, with 16 cases (66.7%) having an AUC greater than 90%, and 9 cases achieving an AUC of over 97%. This result indicates that the classification performance of the proposed hybrid model is highly stable.

**Figure 7:**
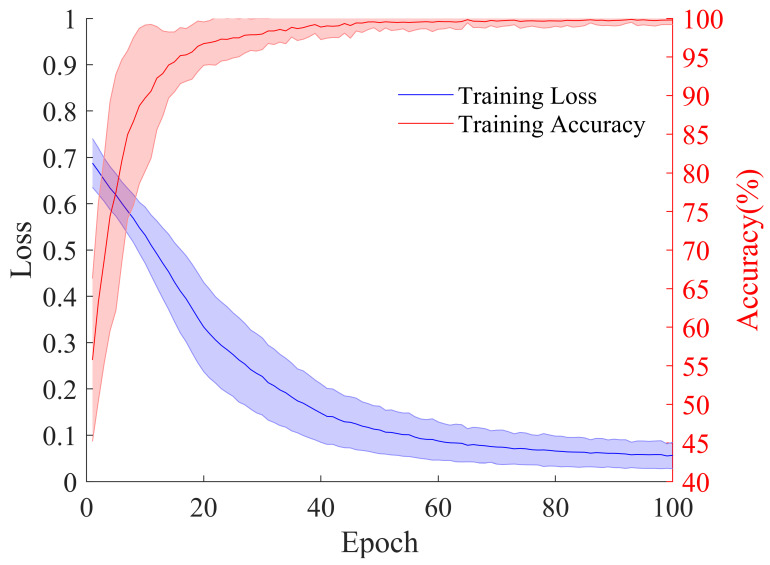
The cross-entropy training loss curve and training accuracy curve for the 24 patients. The shaded area around the curves represents the standard deviation region.

The average cross-entropy training loss and training accuracy curves for the 24 patients are shown in Figure 7. The average cross-entropy training loss and accuracy curves for the 24 patients exhibited a stable convergence trend. The training loss decreased from an initial value of 0.69 to 0.05 within 100 epochs and stabilized, while the training accuracy reached over 99.5% after 50 epochs, ultimately achieving 99.79%. This convergence characteristic indicates that the model achieved efficient feature learning through an end-to-end joint optimization strategy, with no apparent overfitting.

Although high-level results were achieved on this dataset, the performance for certain patients was suboptimal. The higher FDRs observed in some cases can be attributed to the diverse seizure patterns present in the CHB-MIT dataset, as well as significant patient-specific variability. For instance, Patient 13 exhibits a high FDR due to intra-patient variability associated with short-duration seizure events and less distinct seizure features. Meanwhile, Patient 14 has high FDR because of the short duration of seizures, which often manifest as persistent sharp-wave discharges. Similarly, Patient 15 has a high FDR attributed to localized seizures (e.g., in channels like P7-T7), characterized by considerable internal variability in seizure patterns. Moreover, changes in seizure types and brain localizations over time further complicate detection tasks.

### 4.2. Result on SH-SDU Database

To further validate the performance of the proposed model, this study was also tested on the SH-SDU dataset using the same evaluation procedure as in the CHB-MIT dataset. The results, as shown in Table 5, indicate that the segment-based evaluation achieved an average sensitivity of 89.02%, average specificity of 95.46%, and average accuracy of 94.66% across six patients. Furthermore, the specificity and accuracy of all patients exceeded 90%, confirming the feasibility of the model for clinical EEG processing.

**Figure 8:**
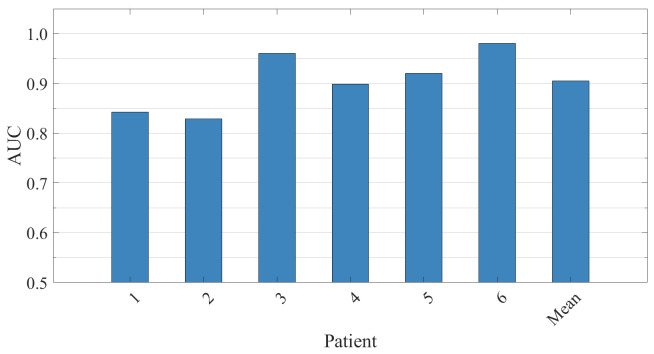
The ROC-AUC results for 6 patients in the SH-SDU dataset are evaluated and presented along with the average values.

Figure 8 presents the AUC bar charts for six patients, further highlighting the model’s high discriminative performance between seizure and normal states. The AUC for all patients exceeds 80%, demonstrating the strong robustness of the model’s feature classification. The results based on the event detection standard are shown in Table 6, where the model successfully identified 93 out of 97 seizure events, achieving an average sensitivity of 97.09%. Additionally, Patient 5 maintained a sensitivity of 94.29% across 35 high-frequency seizures, underscoring the model’s adaptability to variations in seizure frequency. Despite being limited by small-scale training samples and significant EMG/motion artifacts, the model still achieved an average FDR of 2.2137/h. Moreover, it is found in the table that the average latency for all patients is negative, averaging −9 s. This demonstrates that the model is capable of predicting seizures ahead of expert annotations, highlighting its anticipatory ability. These results reinforce the effectiveness of the system in providing timely seizure detection.

**Figure 9:**
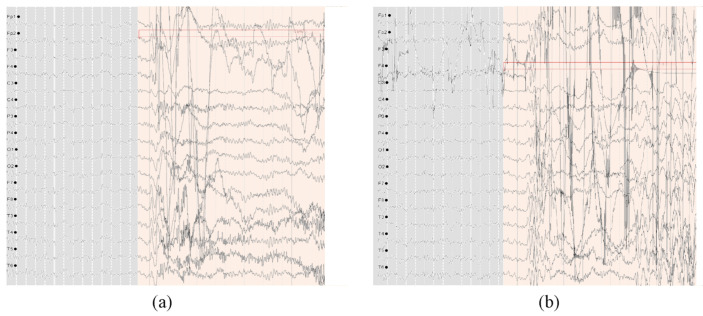
The first two seizure events of Patient 4 in the SH-SDU database. The marked area indicates the seizure annotated by experts. (**a**) The first seizure event with EMG artifacts. (**b**) The second seizure event with continuous EMG artifacts.

Additionally, through examination with medical experts, the high FDR observed in Patients 1 and 4 was due to the effect of EMG noises. Figure 9 illustrates the muscle artifacts in the EEG signals of this patient. Meanwhile, the EEG recordings of Patient 5 exhibit strong variability, and the amplitude of epileptic characteristic waves is relatively low, making it easy to confuse with non-seizure EEG and cause a high FDR.

## 5. Discussion

### 5.1. Ablation Study

#### 5.1.1. Effect of Network Structure

To explore the potential relationship between feature representation and network architecture, we designed four control experiments based on Patient 12 from the CHB-MIT dataset: CNN based on power spectrogram, CNN based on phase spectrogram, ViT based on power spectrogram, and ViT based on phase spectrogram. The experimental results are shown in Table 7. The data clearly exhibit a notable trend in feature-architecture adaptability. In the seizure detection task, the Phase-CNN outperforms the Phase-ViT in terms of AUC, accuracy, and false detection rate, while the Power-ViT surpasses the Power-CNN in these metrics. These findings may suggest that the dynamic non-stationarity of phase features is more suited to CNNs, which extract local features through convolutional kernels, while the global frequency-domain distribution of power spectrogram is more compatible with ViT, which performs cross-frequency global modeling via self-attention. This aligns with the differing roles of phase synchronization and frequency-domain energy features in epileptic signal analysis [49]. Based on this observation, we proposed the hybrid architecture network described above, directing phase features to the CNN branch and power features to the ViT branch based on feature-architecture adaptability. The hybrid architecture network achieves optimal results across AUC, FDR, and accuracy, as shown in Table 7.

#### 5.1.2. Effect of Time–Frequency Methods

Next, we compared the advantages and disadvantages of three time–frequency transformation methods under the unified hybrid architecture. To ensure consistent time–frequency spectrogram sizes as input to the network, the CWT was employed using the same configuration as described in the Method section. The STFT used a Hamming window of length 135 points (approximately 527 ms), with an overlap length of 128 points and a frequency resolution set to 1 Hz. The Gaussian window width adjustment factor for the S-transform was set to 1, with the same frequency range and resolution as CWT. The experimental results are shown in Table 8, where CWT significantly outperforms other methods in the seizure detection task. This phenomenon may be attributed to the multiscale property of CWT and the time–frequency focusing capability of the Morlet wavelet, which is better suited for the transient features of epileptic signals [50]. In contrast, STFT struggles to capture both the high-frequency discharges required by short windows and the low-frequency rhythms needed by long windows [26], leading to blurred transient features. Meanwhile, the S-transform with a fixed Gaussian window adjustment factor may weaken the representation accuracy of phase [51], resulting in the loss of detailed phase synchronization breakdown during seizure periods. This experiment indicates that the CWT is inherently better suited for handling epileptic seizure patterns.

In addition, we compute the model inference time to evaluate the model computational efficiency. The inference times for processing a single 4-s EEG segment are 0.0814 s, 0.0603 s, and 0.0662 s for CWT, S-Transform, and STFT-based models, respectively. Although the inference time for the CWT-based model is slightly higher than that of STFT and S-Transform-based models, this inference time (0.164 s) is significantly smaller than the duration of the segment (4 s). Therefore, the proposed model can fully meet the requirements of real-time seizure detection. More importantly, the CWT-based model outperforms the other two methods in terms of accuracy and AUC. For the offline seizure detection scenarios, the inference time is 3545 s for the proposed CWT-based model to process continuous 24-hour EEG data, which can meet the efficiency requirements for offline seizure detection.

#### 5.1.3. Effect of CWT Wavelet Parameters

To investigate the effects of the central frequency $f_{c}$ and bandwidth $f_{b}$ in CWT, We conducted an ablation study using different $f_{c}$ and $f_{b}$. Experiments with varying values of $f_{c}$ (0.5, 1, 2, 4) and $f_{b}$ (0.5, 1, 2, 4) were conducted. EEG data were transformed using CWT with these different wavelet kernels, and the AUC and FDR of models were reported. The results are depicted in Table 9. The results show that the CWT with $f_{c}=1$ and $f_{b}=1$ yields the highest AUC and the lowest FDR. Hence, we employed these CWT hyperparameter settings in this study.

On the other hand, these parameter settings ($f_{c}=1$ and $f_{b}=1$) were widely adopted in related studies [52,53], and our experiments confirm its optimality in seizure detection applications.

#### 5.1.4. Effect of ViT Branch Depths

To investigate the potential impact of the depth of ViT layer on model performance, we carried out an additional ablation study. Experiments were performed using a hybrid network with 1–3 ViT layers on Patient 12, as shown in Table 10. The result revealed that while AUC for the single-layer ViT configuration was slightly lower than the other configurations, the difference was not significant (by less than 1%). Importantly, the model with a single-layer ViT configuration achieves the lowest FDR, suggesting the advantage of the model with single-layer ViT in real-time seizure detection scenarios.

Furthermore, in terms of parameter scale, the single-layer ViT model (CNN+1ViT) has 249.8 k parameters, whereas adding more layers increases the parameter count significantly—CNN+2ViT has 300.0k parameters, and CNN+3ViT has 350.1 k parameters. Despite the significant increase in parameters, the performance improvement in AUC was not obvious, further reinforcing the choice of the single-layer ViT configuration. This configuration (CNN+1ViT) achieves an optimal balance between performance and computational efficiency.

#### 5.1.5. Effect of Threshold Settings

**Figure 10:**
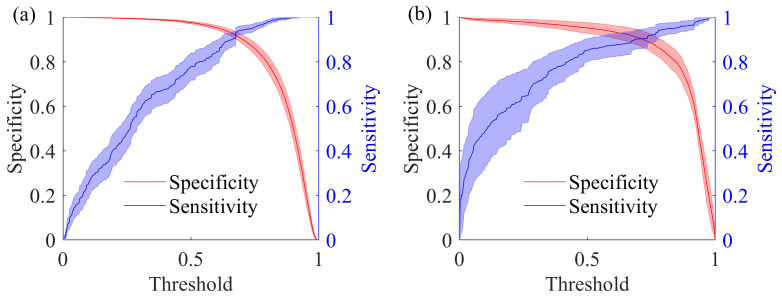
The sensitivity and specificity under different thresholds. (**a**,**b**) The sensitivity and specificity curves for CHB-MIT database and SH-SDU database, respectively. The shaded area indicates the standard error.

To further assess the effect of threshold selection during the postprocessing stage on model performance, we examined how sensitivity and specificity varied across different threshold values. As demonstrated in Figure 10, the threshold that balances sensitivity and specificity is approximately 0.7 for both the CHB-MIT database (see Figure 10a) and the SH-SDU database (see Figure 10b). In clinical practice, clinicians can adjust the threshold based on individual patient characteristics to achieve the most suitable balance between sensitivity and specificity, thus enhancing the system’s accuracy in detecting seizures.

### 5.2. Visualization with t-SNE

**Figure 11:**
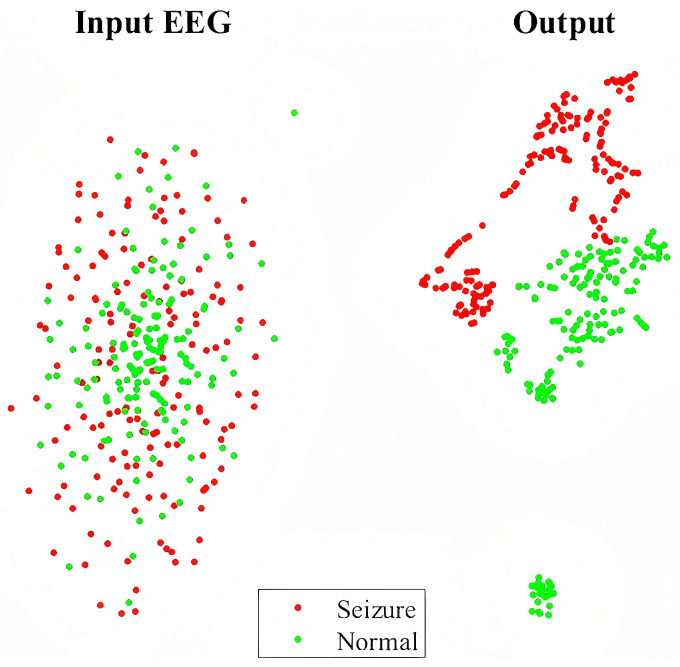
The t-SNE clustering plot of 318 samples from Patient 12 in the CHB-MIT dataset (157 seizure samples and 161 non-seizure samples). The red dots represent the projections of seizure samples, and the green dots represent the projections of normal samples. The plot demonstrates that the clustering of the sample points can be effectively differentiated through the network.

This study visualizes and compares the features extracted from the raw EEG signals and the hybrid network using t-SNE in order to explore the feature enhancement capability of the deep learning model for seizure detection. t-SNE, a nonlinear dimensionality reduction technique [54], optimizes the probability distribution of sample similarities between high-dimensional and low-dimensional spaces, effectively preserving the local structure of the data. It is particularly suited for visualizing the clustering characteristics of high-dimensional data. We applied t-SNE to 318 data samples from Patient 12, as shown in Figure 11. It can be seen that the distribution of seizure and non-seizure samples is mixed with unclear boundaries for raw EEG input, while the separable pattern can be obtained for the output feature of the network, suggesting the great feature extraction ability of the model.

### 5.3. Patient-Independent Performance Evaluation

**Figure 12:**
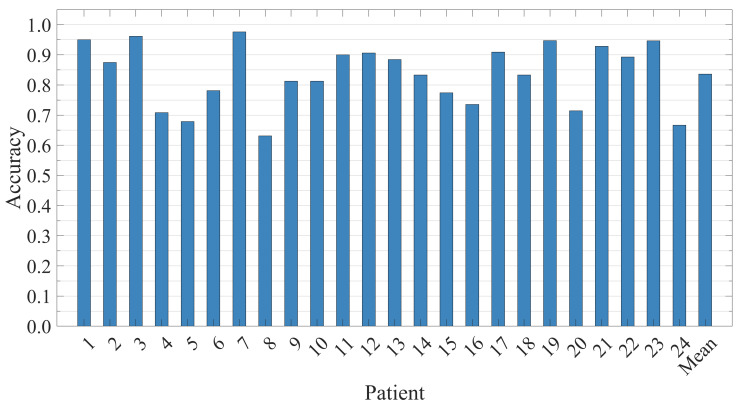
The accuracy for 24 patients in the patient-independent experiment is evaluated and presented along with the average values.

To further evaluate the generalization ability of the proposed model, we performed patient-independent seizure detection assessment across 24 patients in CHB-MIT database using all the training data of the database. Specifically, the leave-one-patient-out evaluation strategy was employed, where one patient’s EEG data served as the testing set while all the other patients worked as the training sets. This partitioning ensures that no data from the same patient appears in both the training and testing sets simultaneously. The results obtained from this partitioning method are shown in Figure 12.

The results indicate that accuracies greater than 90% were achieved on one-third of the patients, with the overall average accuracy exceeding 80%. These findings demonstrate the strong generalizability of our model, suggesting its potential as a patient-independent detection system.

### 5.4. Compared with Existing Methods

Compared to existing methods, Table 11 summarizes the segment-based evaluations, including details of studies from the past five years as well as the current work, covering feature extraction methods, classifiers, and corresponding evaluation metrics. All methods were evaluated on the publicly available CHB-MIT dataset.

As shown in Table 11, compared to existing methods, the proposed study achieves a better balance between sensitivity (98.09%), specificity (98.21%), and FDR (0.31/h). Traditional methods, such as [55], which use EMD-CSP feature extraction with SVM classification, achieved a sensitivity of 97.34%. However, their FDR (0.63/h) is twice that of this study, and the reliance on manual features may result in the loss of transient epileptic waveform characteristics [56,57], which are based on CNN and CNN-Transformer architectures, respectively, achieving accuracies exceeding 96%. However, the former did not validate FDR, and the latter required six times the amount of training data (66,000 s) compared to this study. In recent work, Liu et al. [59] with CosCNN and Li et al. [60] with contrastive learning frameworks show sensitivities close to this study (98.12–98.97%), but their FDRs (0.35–0.69/h) remain higher. The proposed method, through the collaborative design of CWT time–frequency features with CNN-ViT, preserves the non-stationary nature of epileptic signals while utilizing wavelet-domain noise suppression to reduce FDR by 11–55% in high-noise scenarios. Furthermore, the end-to-end architecture eliminates the need for manual feature engineering, providing a more reliable solution for long-term clinical EEG monitoring. The model can serve as an offline seizure detection tool, helping clinicians with highlighting potential seizures, thereby significantly alleviating the workload of clinicians.

## 6. Conclusions

This study confirms the significant value of the phase spectrogram of EEG signals in seizure detection, offering a new analytical dimension for frequency-time feature-based epileptic EEG classification. Addressing the limitations of traditional methods in EEG feature fusion and classification performance, we propose a hybrid CNN-ViT model incorporated with CWT for seizure detection. By jointly extracting time–frequency features from both the power spectrogram and phase spectrogram generated by CWT, and combining the local perceptive ability of CNN with the global dependency-mining advantage of ViT, the system achieves an accurate detection of epileptic seizures. The evaluation results on the CHB-MIT database and our SH-SDU database show that the proposed model achieves ROC-AUCs of 92.91% and 90.52%, sensitivities of 98.09% and 89.02%, specificities of 98.21% and 95.46%, as well as accuracies of 98.45% and 94.66% under segment-based evaluation. Under event-based evaluation, the system achieves sensitivities of 98.95% and 97.09% with FDRs of 0.31/h and 2.21/h. These results not only validate the effectiveness of multimodal time–frequency features and hybrid models but also provide a reliable technical reference for the clinical application of automatic seizure detection. Moreover, the potential use of our model in mobile EEG monitoring and other clinical scenarios is noteworthy, as the lightweight and efficient design of the system makes it well suited for real-time seizure detection applications. Considering the limited clinical EEG data in the current SH-SDU database, we will collect more diverse clinical EEG data to evaluate the generalizability and robustness of our model in the future. We will also focus on multi-center data validation, lightweight deployment, and optimization for real-time monitoring to further improve the clinical applicability and robustness of the system. 

## Figures and Tables

**Table 1:** Details of the CHB-MIT database employed in this study.

Patient–Sex–Age	Seizure Type	Seizure Onset Zone	Total Duration (h)	Mean Seizure Duration (s)	Training Seizure Duration (min)	Training Non-Seizure Duration (min)	Testing EEG Duration (h)
1-F-11	SP, CP	Temporal	40.55	63.15	0.67	3.33	40.48
2-M-11	SP, CP, GTC	Frontal	35.27	57.34	1.35	6.75	35.13
3-F-14	SP, CP	Temporal	38.00	57.43	0.87	4.33	37.91
4-M-22	SP, CP, GTC	Temporal, Occipital	156.07	94.50	0.82	4.08	155.99
5-F-7	CP, GTC	Frontal	39.00	111.60	1.92	9.58	38.81
6-F-1.5	CP, GTC	Temporal	66.74	15.30	1.07	5.33	66.63
7-F-14.5	SP, CP, GTC	Temporal	67.05	108.34	1.43	7.17	66.91
8-M-3.5	SP, CP, GTC	Temporal	20.01	183.80	2.85	14.25	19.72
9-F-10	CP, GTC	Frontal	67.87	69.00	1.07	5.33	67.76
10-M-3	SP, CP, GTC	Temporal	50.02	65.50	0.58	2.92	49.96
11-F-12	SP, CP, GTC	Frontal	34.79	268.67	0.37	1.83	34.75
12-F-2	SP, CP, GTC	Frontal	20.69	36.63	2.15	10.75	20.47
13-F-3	SP, CP, GTC	Temporal, Occipital	33.00	44.59	3.48	17.42	32.65
14-F-9	CP, GTC	Temporal	26.00	21.13	0.23	1.17	25.98
15-M-16	SP, CP, GTC	Frontal, Temporal	40.01	99.60	2.08	10.42	39.80
16-F-7	SP, CP, GTC	Temporal	19.00	8.40	1.15	5.75	18.88
17-F-12	SP, CP, GTC	Temporal	21.01	97.67	1.50	7.50	20.86
18-F-18	SP, CP	Temporal, Occipital	35.63	52.84	0.83	4.17	35.55
19-F-19	SP, CP, GTC	Frontal	29.93	78.67	1.30	6.50	29.80
20-F-6	SP, CP, GTC	Temporal	27.60	36.75	0.48	2.42	27.55
21-F-13	SP, CP	Temporal	32.83	49.75	0.93	4.67	32.74
22-F-9	-	Temporal, Occipital	31.00	68.00	0.97	4.83	30.90
23-F-6	-	Frontal	26.56	60.58	1.88	9.42	26.37
24-/-/	-	-	21.30	31.94	0.42	2.08	21.26
Summary	-	-	979.93	-	30.40	152.02	976.89

Note: GTC = generalized tonic–clonic seizure; CP = complex partial seizures; SP = simple partial seizures.

**Table 2:** Details of the SH-SDU database employed in this study.

Patient–Sex–Age	Seizure Type	Seizure Onset Zone	Total Duration (h)	Mean Seizure Duration (s)	Number of Used Seizures
1-F-28	CP	Temporal, Frontal	20.58	40.53	19–17
2-M-61	CP	Central, Temporal	16.04	220.80	10–8
3-M-34	CP	Temporal, Frontal	12.00	52.20	10–8
4-M-72	CP	Temporal, Frontal	15.56	109.38	29–27
5-M-79	SP	Parietal, Occipital	17.37	68.71	38–35
6-F-38	SP	Temporal	6.00	34.67	3–2
Summary	-	-	87.55	-	109–97

**Table 3:** The segment-based results of the proposed approach on the CHB-MIT database.

Patient	Sensitivity	Specificity	Accuracy
1	100.00%	99.79%	99.86%
2	100.00%	99.96%	99.97%
3	100.00%	99.66%	99.78%
4	82.56%	97.73%	95.58%
5	100.00%	99.89%	99.93%
6	100.00%	99.88%	99.92%
7	96.72%	99.40%	99.05%
8	100.00%	80.13%	86.76%
9	100.00%	99.95%	99.97%
10	100.00%	99.94%	99.96%
11	100.00%	99.80%	99.87%
12	91.77%	98.16%	97.41%
13	87.78%	96.93%	95.92%
14	100.00%	95.57%	97.04%
15	95.26%	97.94%	96.96%
16	100.00%	99.81%	99.87%
17	100.00%	99.89%	99.93%
18	100.00%	99.01%	99.34%
19	100.00%	99.14%	99.43%
20	100.00%	99.20%	99.46%
21	100.00%	99.85%	99.90%
22	100.00%	99.99%	99.99%
23	100.00%	98.31%	98.87%
24	100.00%	97.13%	98.12%
Average	98.09%	98.21%	98.45%

**Table 4:** The event-based results of the proposed approach on the CHB-MIT database.

Patient	Number of Expert- Marked Seizures	Number of Detected Seizures	Sensitivity	FDR (/h)	Latency (s)
1	6	6	100.00%	0.0247	−15.43
2	2	2	100.00%	0.0284	−1.33
3	6	6	100.00%	0.0790	−9.71
4	3	3	100.00%	0.2884	−33.00
5	4	4	100.00%	0.0257	−24.00
6	6	6	100.00%	0.2398	−1.60
7	2	2	100.00%	0.0895	−82.67
8	4	4	100.00%	0.0501	−24.00
9	3	3	100.00%	0.0147	−9.00
10	5	5	100.00%	0.0400	−9.33
11	2	2	100.00%	0.0287	−49.33
12	23	23	100.00%	0.7103	−9.93
13	8	8	100.00%	1.2144	−20.67
14	7	7	100.00%	1.8080	−28.50
15	19	18	94.74%	0.5003	−27.37
16	2	2	100.00%	0.4744	−3.50
17	2	2	100.00%	0.0477	−14.67
18	5	4	80.00%	0.3369	−21.33
19	2	2	100.00%	0.1003	−14.67
20	7	7	100.00%	0.3262	−13.00
21	3	3	100.00%	0.0914	−4.00
22	2	2	100.00%	0	−40.00
23	6	6	100.00%	0.5278	−16.00
24	15	15	100.00%	0.2819	−47.25
Average	144	142	98.95%	0.3054	−21.68

**Table 5:** Segment-based results of the proposed approach on the SH-SDU database.

Patient	Sensitivity	Specificity	Accuracy
1	79.28%	94.04%	90.61%
2	88.80%	97.83%	96.69%
3	99.12%	98.17%	98.63%
4	79.53%	90.50%	90.26%
5	87.38%	92.24%	91.77%
6	100.00%	100.00%	100.00%
Average	89.02%	95.46%	94.66%

**Table 6:** The event-based results of the proposed approach on the SH-SDU database.

Patient	Number of Expert-Marked Seizures	Number of Detected Seizures	Sensitivity	FDR (/h)	Latency (s)
1	17	15	88.24%	3.8416	−13.88
2	8	8	100.00%	0.3137	−8
3	8	8	100.00%	2.0046	−7.6
4	27	27	100.00%	2.7089	−16.97
5	35	33	94.29%	3.8731	−8.56
6	2	2	100.00%	0	0
Average	97	93	97.09%	2.1237	−9.17

**Table 7:** Result comparison on Patient 12 of the CHB - MIT database.

Model	AUC	FDR(/h)	Accuracy
CNN based on power	91.07%	5.5373	93.63%
ViT based on power	91.37%	1.8081	97.30%
CNN based on phase	80.49%	5.8279	92.27%
ViT based on phase	59.02%	20.9708	75.16%
Hybrid	92.57%	0.7103	97.41%

**Table 8:** Results of different time–frequency methods on the CHB-MIT database.

	AUC	Accuracy
CWT	92.57%	97.41%
S-Transform	89.46%	95.37%
STFT	90.18%	96.21%

**Table 9:** Impact of $f_{c}$ and $f_{b}$ parameters on model performance.

$f_{b}$–$f_{c}$	AUC	FDR
1–0.5	92.56%	1.2108
1–2	91.44%	1.1139
1–4	91.69%	0.8233
0.5–1	92.20%	1.0171
2–1	91.79%	1.0655
4–1	91.73%	1.1139
1–1	92.57%	0.7103

**Table 10:** Performance and parameters of the hybrid network with varying ViT layers.

Networks	Number of Parameters	AUC	FDR (/h)
CNN+1ViT	249.8 k	92.26%	0.7104
CNN+2ViT	300.0 k	93.13%	0.8072
CNN+3ViT	350.1 k	93.05%	0.7587

**Table 11:** Performance comparison on different patient-specific seizure detection methods proposed in recent years.

Author	Year	Feature Extraction Method	Classifier	Sensitivity	Specificity	Accuracy	FDR(/h)
Li et al. [55]	2021	EMD+CSP	SVM	97.34%	97.50%	-	0.63
Cimr et al. [56]	2022	Normalization	CNN	97.06%	99.27%	96.99%	-
Zhao et al. [57]	2023	None	CNN+Transformer	97.70%	97.60%	98.76%	-
Liu et al. [58]	2023	WPT+HTBiLGST	MBGWO+FKNN	97.30%	99.48%	99.48%	-
Liu et al. [59]	2024	None	CosCNN	98.12%	99.31%	-	0.69
Li et al. [60]	2024	None	CNN-BiLSTM+Contrastive Loss	98.97%	97.36%	97.36%	0.35
Cao et al. [61]	2025	Time-domain+Nonlinear Features	SVM-REF+CNN-BiLSTM	97.84%	99.21%	98.43%	-
Our work	2025	CWT	CNN+ViT	98.09%	98.21%	98.45%	0.31

## Data Availability

The original contributions presented in this study are included in the article. Further inquiries can be directed to the corresponding authors.
